# Editorial of Special Issue “Embolization Techniques: State of the Art and Future Perspectives”

**DOI:** 10.3390/jcm11175109

**Published:** 2022-08-30

**Authors:** Massimo Venturini, Filippo Piacentino, Andrea Coppola, Federico Fontana

**Affiliations:** 1 Diagnostic and Interventional Radiology Department, Circolo Hospital, ASST Sette Laghi, 21100 Varese, Italy; 2 Department of Medicine and Surgery, Insubria University, 21100 Varese, Italy

Embolization is one of the most important applications in interventional radiology which can be mainly performed using an endovascular approach [1] but also using a percutaneous approach with an intravascular [2] or extravascular target [3]. Embolization is used with the main purpose to obtain a target vessel occlusion [4] or a significant flow reduction as in conventional chemoembolization [5], although it can be also used in the case of a vessel bifurcation to determine an obligatory flow direction, for example in case of gastroduodenal artery coil embolization to favor hepatic intra-arterial chemotherapy [6]. Embolization is routinely performed in many clinical situations including arterial/venous bleeding [7], vascular/lymphatic malformations [8], visceral/renal aneurysms [9,10], endoleaks [11], variceal diseases [12], pre-surgical treatments [13], oncological treatments [14], benign/hypertrophic nodules/organs [15]. Every embolic agent is characterized by points of strengths and weaknesses and can be used alone or combined with another embolic agent to increase its embolic power. For example, in splenic aneurysms coils and liquid embolic agents have been combined to minimize the risk of aneurysm revascularization [16]. In interventional oncology, embolic agents can be used in association with drugs in chemoembolization or in association with percutaneous ablations in combined treatments to enhance therapeutic efficacy. Embolic agents can be released using standard four or five French catheters but often using coaxial microcatheters, particularly in case of tortuous, distal, and/or small caliber vessels. The level of occlusion, proximal or distal to the tip of the delivery catheter, can be chosen according to the vascular disease: for example a proximal coil embolization in case of a visceral aneurysm and a distal particle embolization in case of a bronchial malformation. In the first case a collateral formation is desirable to avoid distal, ischemic complications, in the second case a collateral development can favor a malformation recurrence, and therefore a nidus microvessels occlusion is preferable. Many classifications have been reported to distinguish different embolic agents, for example, mechanical occlusive devices, particulate agents, liquid agents [17], and miscellaneous materials (thrombin, sclerosing agents). Mechanical occlusive devices include coils and plugs that are particularly suitable for proximal embolization. Coils are made up of metallic filaments that roll up within a vessel slowing blood flow, and inducing inflammation and thrombosis. Packing and compaction of coils are very important to obtain a permanent occlusion. Coils are usually sized 20–30% larger than the vessel diameter. Undersized coils risk peripheral migration while oversized coils cannot assume their conformation, minimizing their thrombogenic action. Coils can be differentiated for diameter, length, shape, and in many other ways: 0.035 vs. 0.018 inches for standard catheters and microcatheters, fibered vs. unfibered, pushable vs. detachable. Fibered coils are usually considered more thrombogenic than non-fibered coils. Detachable coils offer the great advantage of retrievability and redeployability compared to pushable coils. Coils can be used not only with other embolic agents but also with stents and balloons for stent-assisted and balloon-assisted coil embolization, respectively. Plugs are device disks made up of a braided nitinol mesh that can be retrieved and readjusted if necessary before their final release. Their thrombogenicity is caused by nitinol mesh and increased by multiple braid layers. Particulate agents, mainly used for distal embolizations, include polyvinyl alcohol particles (PVA), microspheres, gelatin foam sponge, and miscellaneous material (thrombin). Particulate agents are usually mixed with a contrast medium and carried by the blood flow distally to the catheter tip. PVA particles are non-spherical particles, with a size variable from 100 to 1100 microns and capable of a “rasping-action” which promotes aggregation, inflammation, and thrombosis. Unlike PVA, microspheres are spherical, symmetrical, and very precisely sized. The mechanism of thrombogenic action of microspheres is similar to PVA, although the inflammatory reaction is lower. Their size is usually variable from 40 to 1200 microns. Gelatin foam sponge is a temporary embolic agent typically used in pre-operative embolization, post-partum hemorrhage, and conventional chemoembolization [5]. It is a water-insoluble, obtained from purified pork skin gelatin, able to absorb within its interstices blood and other fluids forming a surface for thrombogenesis. Gelatin foam sterile sponge can be cut into several small pieces or mixed with contrast and then injected using a syringe of 2.5–5 mL capacity. Most vessels embolized with Gelfoam are thought to recanalize in a variable time from 3 weeks to 3 months. Liquid embolic agents can usually be divided into adhesive and non-adhesive liquid embolic agents. N-butyl cyanoacrylate (glue) represents the most typical adhesive liquid embolic agent. It polymerizes immediately upon contact with ionic-rich fluids such as blood and it is usually mixed with iodized oil (Lipiodol) to make the material radiopaque and to slow its polymerization time [15]. Before and after N-butyl cyanoacrylate administration, the microcatheter dead space is filled with dextrose solution. Extreme caution is required during glue preparation and delivery to avoid any contact with any ionic solution to prevent premature polymerization. Onyx and Squid are the most used non-adhesive liquid embolic agents, nowadays widely employed also in the extra-cranial district [11]. Onyx and Squid comprise an ethylene vinyl alcohol (EVOH) copolymer dissolved in dimethyl sulfoxide (DMSO) and suspended micronized tantalum powder to provide radiopacity. They required a DMSO-compatible delivery microcatheter. EVOH-based non-adhesive liquid embolic agents need to be prepared with a shaker machine for at least 20 min and before administration, the microcatheter dead space needs to be filled with DMSO. The microcatheter is usually drawn back slowly during liquid embolic injection to avoid its entrapment [16]. Onyx and Squid are similar although Squid is available in three different combinations: 12, 18, 34, characterized by progressively increasing density and viscosity. The less viscous concentration 12, not available for Onyx, can allow a more distal penetration if necessary. Phil (Precipitating Hydrophobic Injectable Liquid) is a more recent DMSO-based liquid embolic agent, initially used in the intra-cranial districts but now also in the non-neurological field. Differently from Onyx and Squid constituted of a mixture with tantalum powder, Phil is composed of a covalently bounded iodine component which does not require a preparation time of 20 min but it is immediately ready and particularly suitable for acute bleeding in emergency [18]. The most used of the miscellaneous materials are probably thrombin, usually used with a percutaneous approach for superficial or visceral pseudoaneurysms [2], and absolute alcohol, a sclerosing agent that has been used for a very long time. It can be used mixed with contrast using a percutaneous or endovascular approach and can determine vasospasm, perivascular necrosis, and thrombosis, for example in arteriovenous malformations. Embolization-related complications can be divided into puncture site, embolization site, and post-procedure [19]. Puncture site complications include hematomas and pseudoaneurysms that can be managed by embolization using an endovascular or percutaneous approach, for example in the case of superficial pseudoaneurysm by percutaneous ultrasound-guided thrombin injection. Embolization site complications include accidental embolization of non-target vessels, distal ischemic complications, dissections, and arterial spasm. Post-procedural complications include post-embolization syndrome, revascularization of the target vessel through collaterals, and infarction of an ischemic organ such as the bowel causing a surgical treatment. In conclusion, many studies published in this Special Issue are innovative approaches to embolization. An innovative approach to embolization can be due to an innovative material, for example, the use of Phil in an emergency [18]. Other innovative applications are due to the use of conventional materials in unconventional districts, such as for example Onyx and Squid in abdominal diseases [11,20] or glue in prostate artery embolization [21], or coils in the hemorrhoidal district [22]. Another innovative approach is the association of two different embolic agents, for example coils and Squid in splenic aneurysms or gastroesophageal varices [9,23]. Innovation in interventional oncology can be due to different particles with peculiar characteristics, for example chemoembolization with degradable starch microspheres in intermediate-advanced HCC [24] or association of conventional materials with an innovative technique as for example the balloon-TACE [25]. 
